# Motivations underlying self-infliction of pain during thinking for pleasure

**DOI:** 10.1038/s41598-022-14775-w

**Published:** 2022-07-04

**Authors:** Andreas B. Eder, Franzisca Maas, Alexander Schubmann, Anand Krishna, Thorsten M. Erle

**Affiliations:** 1 Department of Psychology, Julius-Maximilians-Universität Würzburg, Röntgenring 10, 97070 Würzburg, Germany; 2 Institute for Human Computer Media, Julius-Maximilians-Universität Würzburg, Würzburg, Germany; 3 Department of Social Psychology, Tilburg University, Tilburg, The Netherlands; 4 Present Address: Psychotherapeutische Fachambulanz in Nuremberg, Nuremberg, Germany

**Keywords:** Psychology, Human behaviour

## Abstract

Previous research suggested that people prefer to administer unpleasant electric shocks to themselves rather than being left alone with their thoughts because engagement in thinking is an unpleasant activity. The present research examined this negative reinforcement hypothesis by giving participants a choice of distracting themselves with the generation of electric shock causing no to intense pain. Four experiments (*N* = 254) replicated the result that a large proportion of participants opted to administer painful shocks to themselves during the thinking period. However, they administered strong electric shocks to themselves even when an innocuous response option generating no or a mild shock was available. Furthermore, participants inflicted pain to themselves when they were assisted in the generation of pleasant thoughts during the waiting period, with no difference between pleasant versus unpleasant thought conditions. Overall, these results question that the primary motivation for the self-administration of painful shocks is avoidance of thinking. Instead, it seems that the self-infliction of pain was attractive for many participants, because they were curious about the shocks, their intensities, and the effects they would have on them.

## Introduction

People seem to enjoy many leisure activities, but rarely the mere pursuit of pleasurable thoughts, as Wilson and colleagues^1^ concluded from several studies. When they asked participants to spend several minutes with nothing to do but to think, they did not rate this experience as entertaining and preferred external activities such as listening to music or tampering with one’s own smartphone. In one study, a large proportion even preferred to administer an electric shock to themselves rather than to indulge in their thoughts^2^. Based on these study findings, Wilson and colleagues argued that intentional engagement in thinking is aversive for most people—and hence avoided by seeking a distraction, even if this distraction is known to cause discomfort^1,3^. Fox and colleagues^4^, by contrast, argued in a critical review of Wilson et al.’s research that the preference for one activity (such as surfing the web) does not imply that the non-preferred activity (engaging in pleasant thinking) was necessarily unpleasant. However, if engagement in pleasurable thoughts was entertaining and only less desirable in comparison to a more attractive activity, one must explain why even the self-administration of an electric shock was preferred over thinking. In fact, this experiment provided the strongest clue that voluntary engagement in thinking could be aversive.

## Choosing pain over thinking: the negative reinforcement hypothesis

In the shock study of Wilson and colleagues (^2^, Study 10), 55 undergraduates first rated the pleasantness of several stimuli including a mild electric shock, indicating how much they would pay to (not) experience each stimulus again if they had 5 USD at their disposal. As expected, the majority (76%) indicated that they would pay money to not experience the shock again. Subsequently, they were asked to sit in the chair for 15 min and think about whatever they wanted, with the goal of “entertaining yourself with your thoughts as best as you can.” Participants were also given the option to receive the electric shock again during this period, but that “Whether you do so is completely up to you—it is your choice.” Participants were then left alone, and a computer recorded how many times (if any) they opted to shock themselves.

Analysis showed that 25 participants (45%) opted to shock themselves, with a larger proportion of males (71%) compared to females (26%). The majority shocked themselves 1–2 times, but two outliers even administered 119 and 190 shocks to themselves. Results were robust even when only participants who indicated willingness to pay to avoid future shocks (*n* = 42) and who rated the shock as unpleasant (*n* = 27) were analyzed. Furthermore, there were no significant differences in ratings of enjoyment and boredom between the groups that administered (no) shocks to themselves. One could suspect that participants administered shocks to themselves because they intended to distract themselves from predominantly worrisome and troubling thoughts. However, when the authors analyzed the contents of the generated thoughts during the waiting period, they found no evidence for a prevalence of negative thoughts.

Wilson and colleagues instead explained the self-administration of shocks during the thinking period with the high difficulty of engaging in entertaining thoughts on demand, from which participants could withdraw by administering shocks to themselves. According to their negative reinforcement explanation, self-administration of mild electric shocks was less unpleasant than overcoming the difficulties to engage in thinking, which reinforced the self-infliction of pain.

However, one can also come up with other reasons for why many participants opted to shock themselves. For example, it could be argued that participants were bored by the thinking task, which caused them to seek the thrill of an exciting electric shock^5,6^. People could also seek excitement in the absence of boredom, which is known as sensation-seeking behavior^7^. Sensation seeking could hence explain the self-administration of shocks without making the assumption that thinking was aversive. Furthermore, participants could have shocked themselves out of curiosity, for example, being curious whether the shock would feel the same or about their tolerance for pain. In fact, when Wilson and colleagues^2^ asked participants in post-experimental interviews why they chose to shock themselves, most expressed interest in the quality of the shock and/or its effects^4^.

Further evidence for a potential role of curiosity was provided in a study where participants could kill time by clicking a prank electric-shock pen that was either certain to shock them, certain to not shock them, or uncertain (i.e., could either shock them or not)^8^. Participants clicked pens with uncertain shock administration most often, suggesting that they had a specific desire to resolve uncertainty. They also pressed the pens certain to shock them, albeit at a much lower rate. However, it remains unclear whether curiosity can plausibly account for the shock administrations in the thinking study of Wilson and colleagues, where shock administration following a keypress was certain and had been experienced before.

To summarize, one can hypothesize several motivations for the self-administration of painful electric shocks during a thinking period. The objective of the present study was to subject the negative reinforcement hypothesis to scrutiny. Following the procedure of Wilson and colleagues^2^, we asked participants to sit in a chair without falling asleep and to enjoy their thoughts as best as they could for several minutes. Deviating from the original study, however, we gave them several options for a shock administration during the thinking period. One button triggered a shock with the same (*medium*) intensity as the test shock known from a previous shock intensity adjustment phase. Another button triggered the administration of a *mild* shock with a weaker intensity, while a third button produced a *strong* shock that was more intense than the test shock. A fourth button produced a *random* shock whose intensity varied randomly between strong and mild. Before the thinking period, the contingencies between the keypresses and the shock intensities were thoroughly explained to the participants, but instructions also highlighted that it was completely their choice whether they wanted to receive a shock. Keypresses (shock administrations) were recorded during the thinking period, and after that period, participants rated their enjoyment, excitement, and boredom (among other measures).

We reasoned that if self-administration of shocks is primarily motivated by escape from difficult thinking, as claimed by Wilson and colleagues^2^, then participants should disproportionally prefer to administer the least aversive (mild) shock to themselves. If thrill-seeking is a major drive, then they should disproportionally prefer administration of strong shocks and/or shocks with randomly determined intensities. If people are particularly curious about uncertain shocks^8^, then they should opt for shocks with random intensities.

## Study 1

### Method

#### Transparency and openness

We report how we determined our sample size, all data exclusions (if any), all manipulations, and all measures in the study. Raw data underlying the main findings reported in this paper are available at https://doi.org/10.7910/DVN/IQHACM.

### Participants

Seventy participants (47 females, 23 males; age: *M* = 24.9 years, *SD* = 5.35; *n* = 64 right-handers) volunteered in exchange for a payment. All participants were naïve to the research hypotheses and, after participation, they were excluded from participation in further studies. Participants were recruited via a research participation pool (managed through SONA systems) at the university in order to generate a sample predominantly made up of students, to avoid sampling differences to Wilson and colleagues^2^, who tested mostly undergraduates. Each individual received a payment of 10 EUR per testing hour. Participants provided an informed consent before participation, confirming that they had no cardiovascular diseases or mechanical devices in the body that would threaten the eligibility of receiving electric stimulations. Study protocols were approved by a local research ethics committee (Institute of Psychology, JMU Würzburg, ethics approval number: GZ 2018-15) and all methods were carried out in accordance with the Helsinki declaration on ethical research with human subjects and with guidelines for ethical research with humans from the German Psychological Society (DGPs).

We aimed for samples sizes that were substantially larger than that analyzed by Wilson and colleagues^2^ in the original study (*n* = 42). Sample sizes were planned to have sufficient statistical power (1 − β = 0.80) for the detection of medium-sized effects with a *d* ≥ 0.40 (for a justification of this choice see^9^). Sensitivity analyses performed with GPower 3.1.9.7^10^ confirmed that the sample size *n* = 70 was sufficient for the detection of effects with *d*s >  = 0.30 in the Wilcoxon signed-rank tests of self-administered shocks against zero.

### Apparatus and material

**Figure 1:**
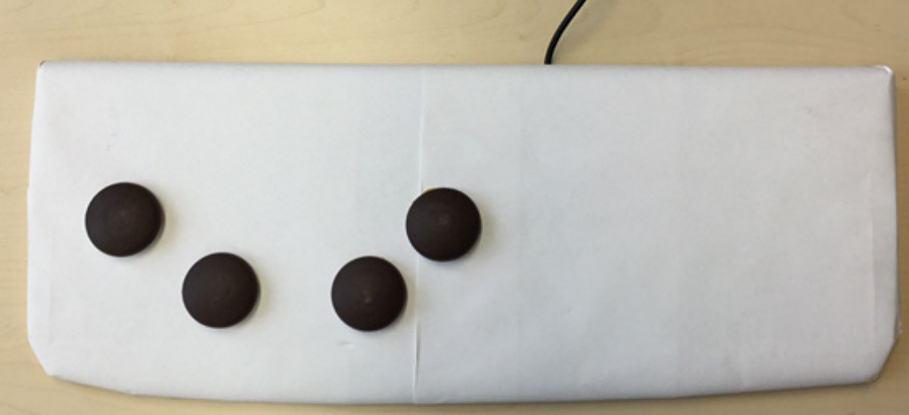
Keyboard device with four buttons used for shock administrations. In Study 4, only a single button was attached to the keyboard.

Stimulus presentation and recording of responses were controlled by a software timer with video synchronization (E-Prime 2.0 Professional; Psychology Software Tools, Inc.). For the administration of a shock, participants used their right hand to press large buttons attached to a computer keyboard that was wrapped in white foil (see Fig. 1).

Electric shocks were delivered by a constant current stimulator (Digitimer DS7A; Digitimer Ltd, Hertfordshire, UK) with an internal frequency of 50 Hz. A bar electrode was attached with an adhesive tape near to the elbow joint of the left forearm. The skin area underneath the electrodes was cleaned with peeling gel and electrodes were filled with a conductive paste. The electric shock was a train of square-wave 2-ms pulses with a 5 ms interpulse interval.

### Shock adjustment procedure

In Wilson et al.’s study (^2^, Study 10), only 76% of the participants indicated that they would pay a hypothetical sum of money to avoid the shock and only 58% gave the test shock an unpleasant rating below the neutral midpoint of the scale (and only 49% indicated both). Thus, it is questionable whether the electric shock was truly unpleasant for the majority of participants.

For the present research, we therefore implemented a staircase procedure^11^ that determined the threshold for a ‘slightly painful’ stimulation on the individual level. After an announcement, the research assistant delivered an electric shock to the participant. Participants rated each shock sensation verbally on a 9-point rating scale with the anchors 0 (‘no sensation’), 1 (‘sensation’), 4 (‘slightly painful’), and 9 (‘maximally tolerable pain’). The research assistant noted the rating of the shock. Shock intensity started with 0 mA and was increased in steps of 0.5 mA until the participant’s intensity rating reached a score 4 or higher. For the next calibration cycle, the shock intensity was first increased by an additional 0.5 mA and then stepwise decreased by 0.5 mA until the participant’s intensity rating was below 4. Two additional calibration cycles followed with start values 0.5 mA below (calibration with increasing intensities) or above the last value (calibration with decreasing intensities), resulting in a total of three calibration cycles each with ascending and descending arms. After the last calibration cycle, intensities with a rating score 4 were averaged and the intensity was additionally increased by 30% for the actual experiment. The minimum intensity for the final test shock was set to 1 mA and the maximum intensity could not exceed 5 mA for ethical reasons^12^. Intensities of adjusted test shocks were: *M* = 2.25 mA (*SD* = 1.27) in Study 1; *M* = 2.77 mA (*SD* = 1.39) in Study 2; *M* = 2.85 mA (*SD* = 1.37) in Study 3; *M* = 2.39 mA (*SD* = 1.23) in Study 4.

Intensities of strong and mild electric shocks administered during the thinking period were manipulated by increasing or decreasing the duration of the shocks, respectively. The medium shock always was the test shock with ten electric pulses. For a strong shock, the duration was increased from 10 to 20 electric pulses, whereas for a mild shock the duration was decreased to 6 electric pulses (both with a 5 ms interpulse interval). Research demonstrated that increased duration of punishment has analogous aversive effects compared to increased intensity of punishment^13^. Furthermore, a previous study using the same duration manipulation confirmed that the strong shock, relative to the mild shock, evoked more fear and more physiological arousal as indexed by a greater change in skin conductance levels^11^.

### Testing procedure

Participants were tested individually in a sparsely furnished laboratory room. Personal belongings that could be used for distraction were locked away. After providing an informed consent, the intensity of the test shock was adjusted using the procedure described above. Then, participants rated their current mood on a scale from ‘very bad’ (−4) to ‘very good’ (+ 4). Subsequently, the following information appeared on the computer screen: “Next follows a 15 min waiting period. Your task for this period is to entertain yourself with your thoughts. You can think about anything you want.” Instructions then stated that pressing the button(s) was allowed during the waiting period, and that each button press would administer an electric stimulation with a different intensity:

The button “FAMILIAR shock” would produce the test shock that was experienced in the previous shock adjustment phase. The button “WEAKER shock” would produce an electric shock that was weaker, and the button “STRONGER shock” a shock that was stronger than the test shock. The button “RANDOM shock” would produce a shock whose intensity could be stronger or weaker than the test shock.

Four labelled squares in horizontal arrangement were displayed on the screen during the thinking period that informed the participant about the function of each key. Assignment of the shock intensities to the four buttons was randomized. Instructions explicitly highlighted that a button press is not necessary: “You can press buttons a single time, several times, or not at all. This decision is completely yours! Entertain yourself with your thoughts. See you later!” The research assistant then left the room and returned after 15 min.

After the thinking period, participants again rated current mood, pleasantness (−4 very unpleasant, 0 neutral, + 4 very pleasant), boredom (1 not at all boring, 5 neutral, 9 very boring), and excitement (1 not at all exciting, 5 neutral, 9 very exciting) during the thinking period. Then, they were asked to describe their motivational reasons for a button press in own words on a sheet of paper. Subsequently, they were asked to complete the following personality questionnaires: (1) The subscales “Openness to Experience” (Open; 10 items, Cronbach’s α = 0.76), “Neuroticism” (Neuro; 10 items, α = 0.90), and “Need for Achievement” (nAch; 6 items, α = 0.82) of the German Big Five Personality Inventory (B5T)^14^. (2) The German version of the “Need for Cognition Scale” (NFC, 16 items, α = 0.83)^15^. (3) The subscales “Need for Stimulation” (NS, α = 0.82) and “Avoidance of Rest” (AR, α = 0.82) of the German “Need Inventory of Sensation Seeking” (NISS, 17 items)^16^. (4) The “Impression Management Scale” (IMS, 17 items, α = 0.82)^17^. (4) The subscale “Bravado” (2 items) of the German Narcissistic Personality Inventory (NPI-d)^18^ and a German translation of the Single Item Narcissm Scale (SINS; “To what extent do you agree with this statement: I am a narcissist.”)^19^. Finally, they were thanked, debriefed, paid, and dismissed.

### Data-analytic approach

Dependent measures of main interest were the proportion of participants who opted to shock themselves during the thinking period and the numbers of self-administered shocks as a function of the shock type (mild, medium = familiar, strong, random). Proportions were compared using z-tests with continuity correction. Counts of self-administered shocks as a function of shock intensity were compared using a related-samples Friedman test, because they violated normal distribution even after transformation. Follow-up tests were conducted with Wilcoxon signed-rank tests that were Bonferroni-corrected for multiple testing. Data were analyzed before and after removal of outlier values in the total number of self-administered shocks. Following the recommendation of Tukey^20^, a value was identified as outlier if it was equal to or larger than 1.5 interquartile ranges above the third quartile of the data distribution. Changes in statistical results after outlier removal are noted in the report.

Timing of the button presses during the waiting period was only recorded in Study 3 and Study 4. For explorative analyses, rates of responding in these studies were plotted as a function of time in minutes and analyzed for statistical trends (linear, cubic, and quadratic) in analyses of variance (ANOVA) with *Waiting Time* (each minute of the waiting period; within) as factor.

Participants rated their mood before and after the thinking period (after minus before = mood change), feelings of pleasantness, boredom, and excitement during the thinking period (among other measures). These ratings were analyzed for correlations with frequencies of self-administered shocks. Exploratory correlational analyses between personality measures and self-administration of shocks are reported in the supplement.

Aggregated across all four studies, men (*n* = 81) or women (*n* = 173) did not administer more shocks to themselves and there were no significant gender differences in the proportions who opted for a self-administration of shocks (see the supplement to this article for corresponding analyses). Therefore, data were collapsed across genders for the analyses reported next.

### Results

#### Self-administration of electric shocks

**Figure 2:**
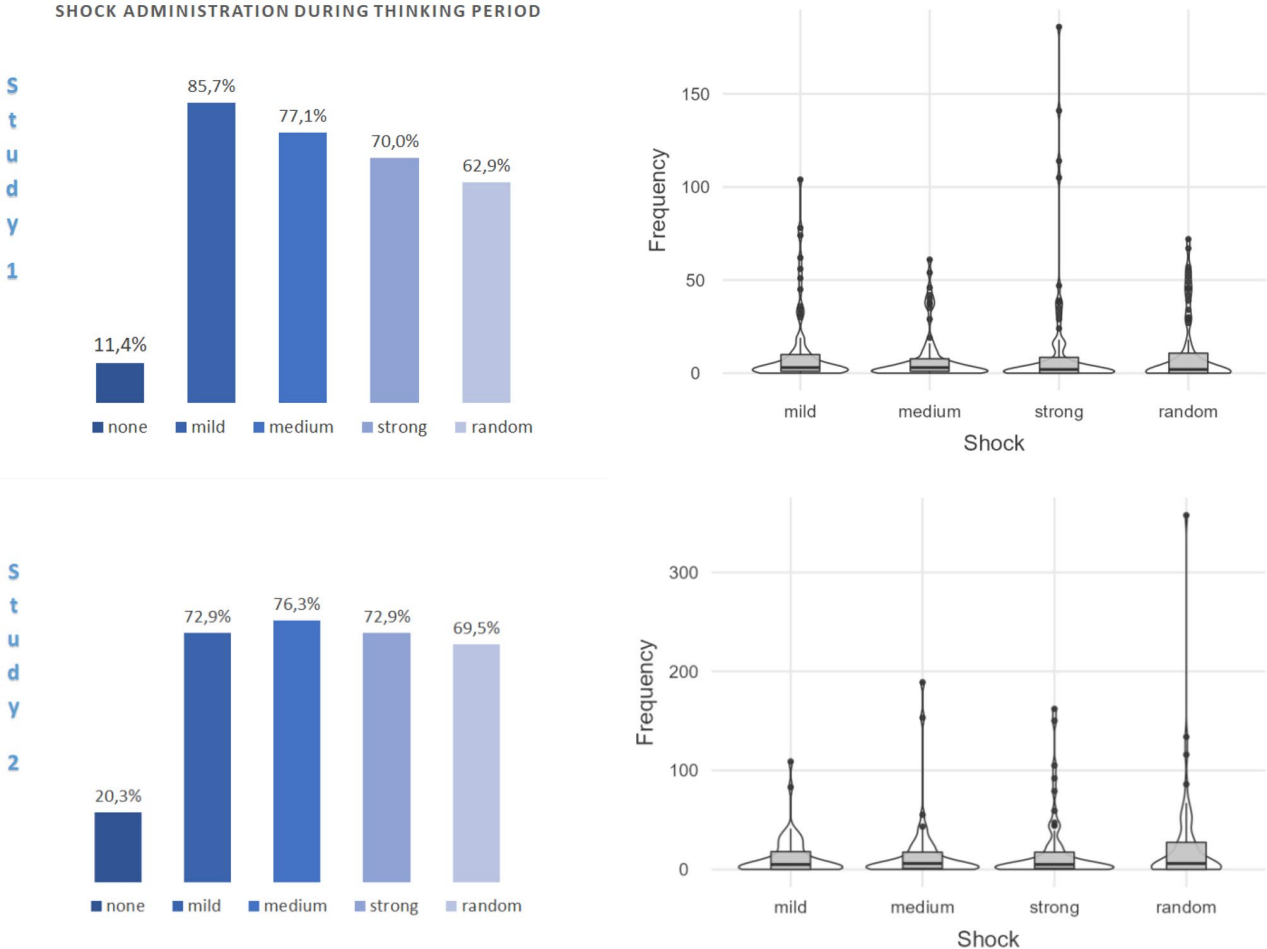
Left panel: proportions of participants who administered shocks to themselves during the thinking period. Note that the ‘none’ bar is mutually exclusive with the others; however, a participant can be counted in all other bars simultaneously. Right panel: violin plots with box plots of the frequencies of self-administered shocks as a function of shock intensity.

Figure 2 shows the results. Sixty-two out of 70 participants (88.6%; 87% males, 89.4% of the females) opted to administer a shock to themselves. The majority of participants administered all of the different types of shock to themselves.

The median of the total numbers of self-administrated shocks during the thinking period was *Mdn* = 10; however, inter-individual differences were very large (range: 314). Median values of self-administered shocks were in the order: mild (*Mdn* = 3, *IQR* = 9.5) = medium (*Mdn* = 3, *IQR* = 7.5) > strong (*Mdn* = 2, *IQR* = 9.75) = random (*Mdn* = 2, *IQR* = 11.5). The effect of shock type (mild, medium, strong, random) on the numbers of self-administered shocks was significant in a related-samples Friedman test, χ^2^(3) = 13.694, *p* = 0.003. Post hoc analysis with Bonferroni-corrected Wilcoxon signed-rank tests revealed fewer administrations of medium shocks relative to mild shocks, *z* = 3.04, *p* = 0.012, *r* = 0.36. Other differences were not significant with *p*s > 0.27. One-sample Wilcoxon signed-rank tests confirmed that the median values were significantly greater than zero for administrations of mild, *z* = 6.75, *p* < 0.001, *r* = 0.80, medium, *z* = 6.40, *p* < 0.001, *r* = 0.76, strong, *z* = 6.11, *p* < 0.001, *r* = 0.73, and randomly determined shock intensities, *z* = 5.78, *p* < 0.001, *r* = 0.69. After removal of 9 outlier values (*n* = 61), results with statistical significance were the same except that the difference between mild (*Mdn* = 3) and strong shocks (*Mdn* = 1) was also significant in the Bonferroni-corrected post hoc Wilcoxon signed-rank test, *z* = 3.01, *p* = 0.018, *r* = 0.36.

#### Ratings of the thinking period

Mood was rated on the scale from −4 to + 4, and was non-significantly better before (*M* = 1.84, 95%CI [1.48, 2.20]) than after the thinking period (*M* = 1.73, 95%CI [1.37, 2.08]), *t*(69) = 1.34, *p* = 0.184, *d*_*z*_ = 0.16 (95%CI [−0.08, 0.39]). Participants rated the thinking period as slightly pleasant (*M* = 0.89, 95%CI [0.47, 1.30]), moderately boring (*M* = 4.76, 95%CI [4.28, 5.23]), and as not exciting (*M* = 3.07, 95%CI [2.66, 3.49]). Correlational analyses revealed statistically significant positive correlations between numbers of shock administration and ratings of boredom and excitement (see Table 1).

**Table 1:** Spearman’s rank-based correlations between mood change, ratings of the thinking period, and counts of self-administered shocks during the thinking period.

	1	2	3	4	5	6	7	8
1. MOOD	–							
2. PLEASANTNESS	0.235*	–						
3. BOREDOM	−0.096	−0.545***	–					
4. EXCITEMENT	0.138	0.136	−0.098	–				
5. Mild	0.009	−0.055	0.337**	0.324**	–			
6. Medium	0.062	−0.117	0.322**	0.231	0.898***	–		
7. Strong	0.012	−0.135	0.242*	0.287*	0.791***	0.861***	–	
8. Random	0.110	−0.141	0.244*	0.302*	0.818***	0.836***	0.843***	–
9. Total count	0.055	−0.108	0.313**	0.298*	0.944***	0.965***	0.902***	0.901***

*p < .05, **p < .01, ***p < .001.

### Discussion

A large majority of participants opted to administer an unpleasant electric shock during the thinking period. This result replicates Wilson and colleagues (^2^ Study 10), and the proportion was even larger than in the original study (88.6% vs 45%). Even more important for the present research question, participants administered the familiar (medium) shock least often, with no differences in self-administrations of mild, strong, and random shocks. The large number of self-administered strong shocks is at odds with the negative reinforcement hypothesis which predicted a preference for the least aversive response option (mild shock). In contrast, correlations showed a positive relationship with ratings of boredom and excitement, which fits better to an explanation with sensation seeking and/or exploration. It is possible that participants were curious of shocks with mild, strong, or random intensities because, in contrast to the medium shock, these intensities were not experienced before the waiting period. This explanation was tested in a second experiment.

## Study 2

Study 2 was identical with Study 1 with the single change that each shock type was experienced in a familiarization phase immediately prior to the thinking period. If participants administered shocks to themselves because they were curious about the quality of the shocks, this curiosity should (partly) be satisfied by the familiarization phase in Study 2. Hence, this explanation predicted a lower proportion of participants administering a shock to themselves in Study 2 compared to Study 1.

### Method

#### Participants

Fifty-nine participants (45 females; age: *M* = 25.4 years, *SD* = 4.74; *n* = 54 right-handers) volunteered in exchange for a payment. A sensitivity analysis for a one-tailed z-test of independent proportions with continuity correction showed that samples sizes were sufficiently powered (1-beta = 0.80) for the detection of a proportion difference ≥ 19%.

#### Apparatus, materials and procedure

Study 2 differed from Study 1 in the inclusion of a familiarization phase before the thinking period. In this phase, instructions guided the participant to press each button once for an experience of the associated shock intensity. After that, the thinking period started. The open-ended questionnaire was removed because of time constraints.

### Results

#### Self-administration of electric shocks

Figure 2 summarizes the results. In Study 2, 47 of 59 participants (79.7%; 85.7% males, 77.8% females) administered shocks to themselves. A one-tailed *z*-test with continuity correction indicated that this proportion was significantly smaller than the proportion 88.6% observed in Study 1 without familiarization phase, *z* = −1.95, *p* = 0.026, *r* = 0.25.

The median of the total number of self-administered shocks during the thinking period was *Mdn* = 22; however, inter-individual differences were again extremely large with a maximum count of 605 keypresses (40.3/min) for one individual. Median values of self-administered shocks were in the order: medium (*Mdn* = 6, *IQR* = 17) > mild (*Mdn* = 5, *IQR* = 19) = random (*Mdn* = 5, *IQR* = 27) > strong (*Mdn* = 4, *IQR* = 19). The effect of shock intensity (mild, medium, strong, random) on the number of self-administered shocks was not significant in the Friedman test, χ^2^(3) = 7.054, *p* = 0.070. One-sample Wilcoxon signed-rank tests of median values against zero revealed significant self-administrations of shocks with mild, *z* = 5.71, *p* < 0.001, *r* = 0.74, medium, *z* = 5.85, *p* < 0.001, *r* = 0.76, strong, *z* = 5.68, *p* < 0.001, *r* = 0.74, and random intensities, *z* = 5.58, *p* < 0.001, *r* = 0.73. Removal of four outlier values (n = 55) in the total number of self-administered shocks did not change the statistical results.

#### Ratings of the thinking period

Mood was non-significantly better before (*M* = 2.02, 95%CI [1.71, 2.33]) than after the thinking period (*M* = 1.90, 95%CI [1.54, 2.25]), *t*(58) = 1.07, *p* = 0.290, *d*_*z*_ = 0.139 (95%CI [−0.12, 0.40]). Participants rated the thinking period as slightly pleasant (*M* = 0.88, 95%CI [0.44, 1.33]), moderately boring (*M* = 4.93, 95%CI [4.34, 5.52]), and as unexciting (*M* = 3.03, 95%CI [2.54, 3.53]). Correlational analyses revealed statistically significant positive correlations between frequencies of shock administration and ratings of excitement (see Table 2).

**Table 2:** Spearman’s rank-based correlations between mood change, ratings of the thinking period, and counts of self-administered shocks during the thinking period.

	1	2	3	4	5	6	7	8
1. MOOD	–							
2. PLEASANTNESS	0.176	–						
3. BOREDOM	−0.061	−0.765***	–					
4. EXCITEMENT	−0.068	0.232	−0.354**	–				
5. Mild	0.132	−0.160	0.100	0.307*	–			
6. Medium	0.077	−0.184	0.136	0.372**	0.936***	–		
7. Strong	0.030	−0.154	0.079	0.367**	0.892***	0.947***	–	
8. Random	0.187	−0.204	0.183	0.331*	0.855***	0.866***	0.879***	–
9. Total count	0.073	−0.188	0.141	0.378**	0.941***	0.973***	0.969***	0.933***

*p < .05, ** *p* < .01, *** *p* < .001.

### Discussion

A large proportion of participants again opted to administer shocks to themselves. However, in this study the proportion was significantly smaller compared to Study 1 (79.7% vs 88.6%). The inclusion of a familiarization phase with administrations of each shock type before the thinking period, hence appeared to have reduced the willingness to administer a shock to themselves. This reduction is in line with an explanation that participants were curious of novel shocks and how they would feel. In addition, correlational analyses revealed a relationship with ratings of excitement, suggesting an involvement of sensation seeking. The large numbers of self-administered strong shocks is however difficult to reconcile with the negative reinforcement explanation.

## Study 3

A potential explanation for why participants did not prefer the mild shock over more painful shocks is that the reduced pain intensity was not large enough for a negative reinforcement of the behavior. Study 3 therefore tested the negative reinforcement hypothesis with the provision of a response option (response button) that did not generate an unpleasant sensation (i.e., electric shock). This innocuous response option was made available in addition to buttons that produced mild, medium, and strong shocks. If avoidance of thinking engagement is the primary motivation to seek distraction with an external activity, participants should opt for the least aversive distraction, which was keypressing for no shocks.

### Method

#### Participants

Thirty-six students (21 females; age: *M* = 29.1 years, *SD* = 9.54; 34 right-handers) volunteered for participation. Sample size was smaller given the large effects in the Wilcoxon signed-rank tests in Study 1 and 2 (with *ds* ≥ 1.91). In this study, we also asked the participant in the post-experimental questionnaire whether he or she understood that a button press during the thinking period was optional and therefore not necessary. Two participants indicated low knowledge of the instruction that a keypress was not demanded from them (ratings 1 and 3, respectively, on a scale from 1 to 9). These two data sets were removed.

#### Apparatus and procedure

Materials and procedures were the same as in Study 2 with the major change that the random shock button was replaced with a button that produced no shock. Participants additionally rated the enjoyment of their thoughts from −4 (very unpleasant) to + 4 (very pleasant), and indicated whether they understood the instruction that a keypress during the thinking period was not necessary (1 = ‘No, I believed a button press was necessary, 9 = ‘Yes, that was clear to me’). The time-consuming personality questionnaires were removed from this and the subsequent study.

### Results

#### Self-administration of electric shocks

**Figure 3:**
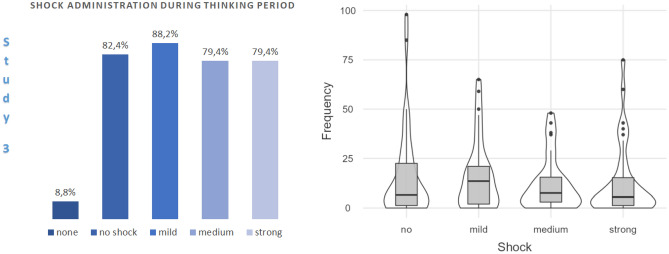
Proportion of participants (*n* = 34) who administered shocks to themselves during the thinking period (left panel) and a violin plot with box plots of the frequencies of self-administered shocks as a function of shock type (right panel). Note that the ‘none’ bar in the left panel is mutually exclusive with the others; however, a participant can be counted in all other bars simultaneously.

Nearly all participants (91.2%) pressed a button during the waiting period, and 88.2% (73.3% of the males, 100% of the females) administered shocks to themselves (see Fig. 3).

The median of the total number of keypresses during the thinking period was *Mdn* = 42.5 (range: 251); the median total number of self-administered shocks was *Mdn* = 28 (range: 162). Median numbers of keypresses were in the order: mild (*Mdn* = 13.5, *IQR* = 20.25) > medium (*Mdn* = 7.5, *IQR* = 13.5) > no shock (*Mdn* = 6.5, *IQR* = 13.25) > strong (*Mdn* = 5.5, *IQR* = 16.25). The effect of shock administration (none, mild, medium, strong) on the numbers of keypresses was significant in the Friedman test, χ^2^(3) = 12.97, *p* = 0.005. Post hoc analysis with Bonferroni-corrected Wilcoxon signed-rank tests revealed more self-administrations of mild shocks than medium shocks, *z* = 3.17, *p* = 0.012, *r* = 0.54, and strong shocks, *z* = 2.90, *p* = 0.024, *r* = 0.50. Other differences were not significant with *p*s = 1. One-sample Wilcoxon signed-rank tests against zero confirmed significant production of keypresses generating no shock, *z* = 4.62, *p* < 0.001, *r* = 0.79, mild shocks, *z* = 4.78, *p* < 0.001, *r* = 0.82, medium shocks, *z* = 4.54, *p* < 0.001, *r* = 0.78, and strong shocks, *z* = 4.54, *p* < 0.001, *r* = 0.78. Removal of two outlier values in the total number of button presses did not change these results.

**Figure 4:**
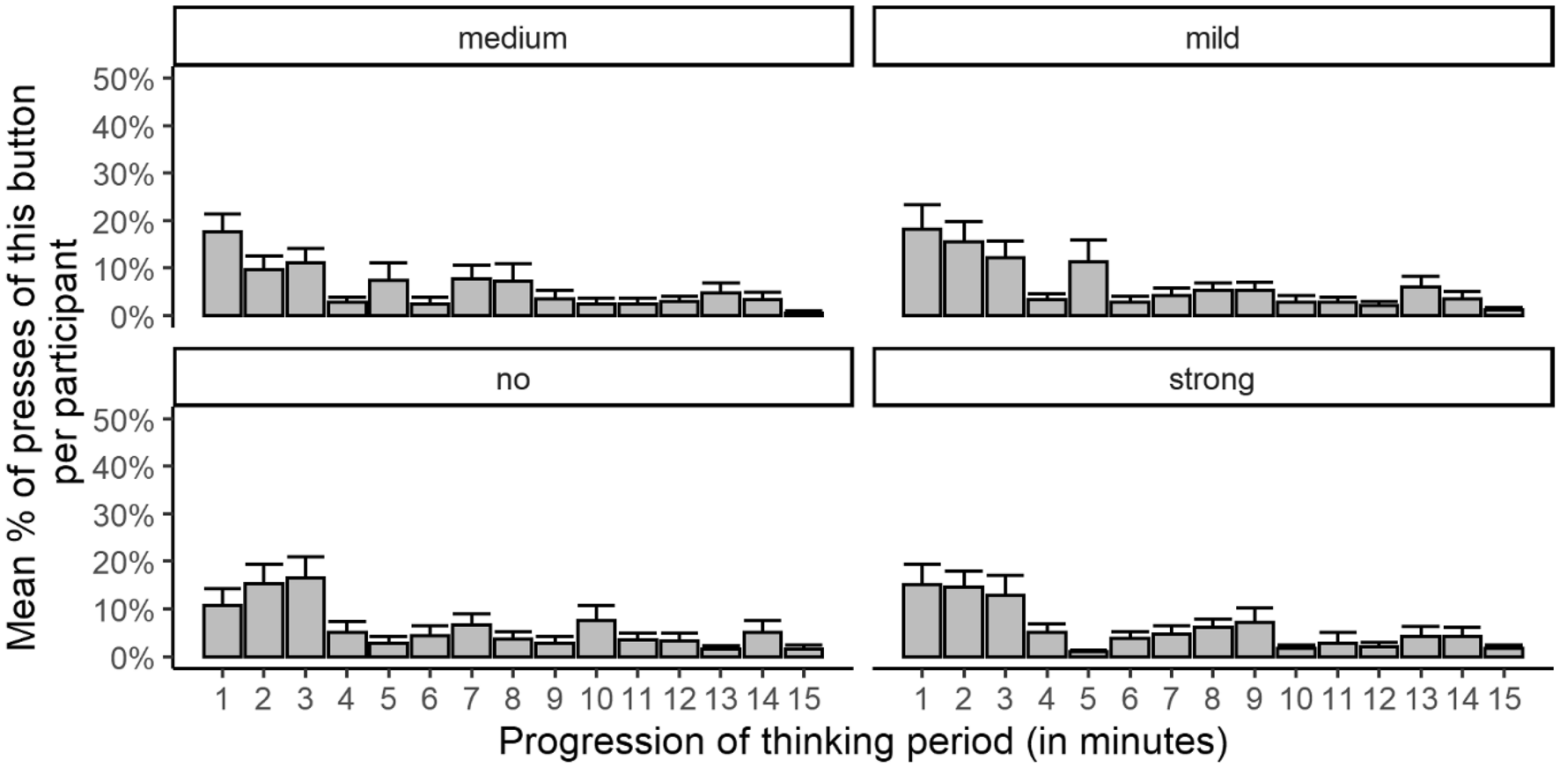
Rate of button presses as a function of time. Error bars show the standard error. Subjects without button presses and two subjects with outlier values were not included in the counts (valid *n* = 30).

Figure 4 shows the rate of keypressing as a function of time. Participants waited 46.7 s (*SD* = 63.7) on average before the first keypress and they pressed buttons most often during the first minutes of the waiting period. Statistical trend analyses of proportional response rates per minute for each shock type in a Bonferroni-adjusted ANOVA with *Waiting Time* (15: each minute of the waiting period, within) as factor revealed significant linear trends for button presses generating no shock, *F*(1, 29) = 14.94, *p* = 0.002, ${\upeta }_{\mathrm{p}}^{2}$=0.340; mild shocks, *F*(1, 29) = 26.98, p < 0.001, ${\upeta }_{\mathrm{p}}^{2}$=0.482; medium shocks, *F*(1, 29) = 19.08, *p* < 0.001, ${\upeta }_{\mathrm{p}}^{2}$=0.397; and strong shocks, *F*(1, 29) = 15.70, *p* = 0.002, ${\upeta }_{\mathrm{p}}^{2}$=0.351. Quadratic and cubic trends were not significant with *p*s > 0.05.

#### Ratings of the thinking period

Mood was significantly better before (*M* = 1.68, 95%CI [1.34, 2.02]) compared to after the thinking period (*M* = 1.24, 95%CI [0.82, 1.65]), *t*(33) = 2.60, *p* = 0.014, *d*_z_ = 0.445 (95%CI [0.09, 0.80]). Participants rated the thinking period as slightly pleasant (*M* = 0.32, 95%CI [-0.22, 0.86]), moderately boring (*M* = 4.88, 95%CI [4.13, 5.63]), and as not exciting (*M* = 3.03, 95%CI [2.38, 3.68]). Thoughts were rated slightly positively *M* = 1.15 (95%CI [0.68, 1.62]) on a scale from -4 (very unpleasant) to + 4 (very pleasant). Correlational analyses did not reveal a significant relationship with the numbers of keypresses (see Table 3).

**Table 3:** Spearman’s rank-based correlations between mood change, ratings of the thinking period, and counts of keypresses producing (no) shocks during the thinking period.

	1	2	3	4	5	6	7	8	9
1. MOOD	–								
2. PLEASANTNESS	0.389*	–							
3. BOREDOM	−0.332	−0.666***	–						
4. EXCITEMENT	0.052	−0.022	−0.183	–					
5. ENJOYMENT	0.274	0.453**	−0.621***	−0.140	–				
6. No shock	0.190	0.407*	−0.303	−0.123	0.107	–			
7. Mild	0.086	0.240	−0.131	−0.007	0.144	0.648***	–		
8. Medium	0.175	0.228	−0.025	−0.147	0.168	0.502**	0.913***	–	
9. Strong	0.155	0.283	−0.101	−0.066	0.142	0.452**	0.894***	0.919***	–
10. Total count	0.170	0.301	−0.135	−0.073	0.130	0.704***	0.972***	0.925***	0.916***

*p < .05, **p < .01, ***p < .001.

### Discussion

Although participants had an innocuous response option available for distraction from the thinking task, a large majority still opted to administer (strongly) unpleasant shocks to them. This behavior is at odds with the negative reinforcement explanation, by which participants should have selected the least aversive response option for distraction from the (even more aversive) thinking task. Rather, it seems that shocking oneself was attractive for many participants, irrespective of the nature of the thinking task. This assumption was scrutinized more closely in a fourth study.

## Study 4

Wilson and colleagues argued that deliberately thinking for pleasure is cognitively taxing and difficult—and hence avoided by seeking distractions^1,3^. People can however be assisted in the generation of thoughts. Specifically, Westgate and colleagues^21^ designed a thinking aid procedure that makes it easier to engage in pleasurable thoughts. Participants in their study were first asked to write down pleasurable thoughts that were presented during the thinking period as prompts of a pleasurable thought. Results showed that participants found it easier to engage in pleasurable thinking and enjoyed the thinking period more compared to a control condition without a “thinking aid.”

In Study 4, we implemented an analogous thinking aid procedure to assist participants in the generation of thoughts during the waiting period. Participants were randomly assigned to one of three conditions: (1) a condition with a thinking aid for the generation of pleasant thoughts; (2) a condition with a thinking aid for the generation of unpleasant thoughts; (3) no thinking aid (control condition). In this experiment, only one button was available, and participants could administer a slightly painful shock to themselves by pressing this button. If the thinking aids facilitate engagement in thinking, participants should be less inclined to administer painful shocks to themselves in the thinking aid conditions relative to the control condition. Furthermore, if escape from negative thought is the primary motivation for the self-administration of shocks, they should shock themselves most often in the unpleasant-thought condition and least often in the pleasant-thought condition.

### Method

#### Participants

*N* = 94 volunteers (*n* = 64 female; age: *M* = 27.1 years, *SD* = 9.21; *n* = 87 right-handers) were randomly assigned to the pleasant thinking aid (*n* = 32), unpleasant thinking aid (*n* = 31), and no thinking aid (*n* = 31) conditions. Data from three participants (two in the pleasant and one in the unpleasant conditions) were removed from analyses based on poor understanding of the instruction that a button press was not necessary. Sensitivity analyses confirmed that sample size (*n* = 91) was sufficient for the detection of a medium sized effect (*w* = 0.32) in the chi-square tests of group differences.

#### Procedure

There was only a single button available during the thinking period and a press administered the test shock to the participant. Before the thinking period, participants were told to administer the test shock to themselves for an experience of the shock. Depending on the thinking aid condition, participants were asked either to list eight pleasant thoughts or fantasies that they would enjoy thinking about (pleasant thinking aid condition), eight unpleasant thoughts or worries that they would not enjoy thinking about (unpleasant thinking aid condition), or to answer a series of simple trivia questions (e.g., “Name a country in Asia”; no thinking aid/control condition). During the thinking period, the generated topics were displayed one at a time on the computer screen, and participants were instructed to think about the currently displayed thought or hope/worry; in the control condition, participants were reminded of thinking without presentation of a topic. Following the procedure of Westgate and colleagues^21^, a generated topic was displayed on the computer screen at every 45 s, reducing the total duration of the thinking period to 6 min. After the thinking period, participants rated their current mood; pleasantness, enjoyment (1 = ‘not at all entertaining, 9 = ‘very entertaining’), and boredom during the thinking period; the frequency of mind-wandering (1 = ‘ not at all, 9 = ‘very frequent’), and the difficulty of thinking during the waiting period (1 = ‘not at all difficult’, 9 = ‘very difficult’). In addition, they were asked whether they understood the instruction that a button press was not necessary (yes/no).

### Results

#### Self-administration of electric shocks

**Figure 5:**
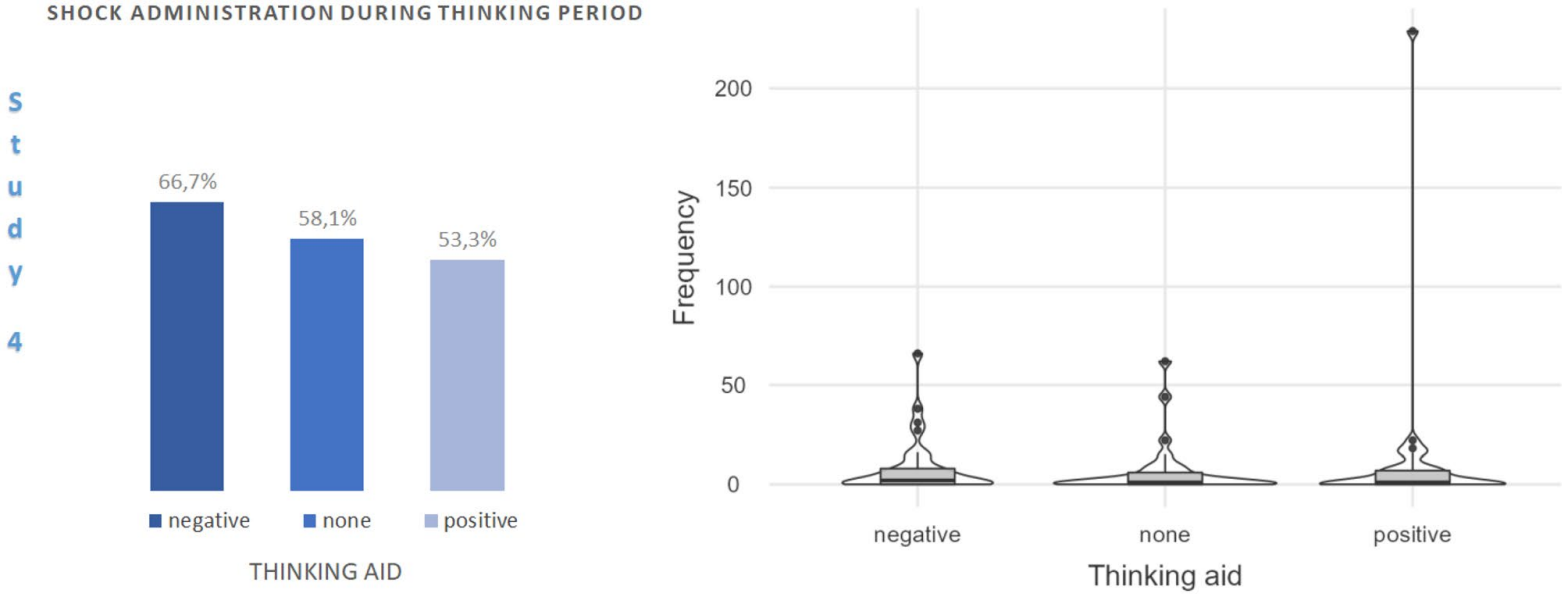
Self-administration of shocks in each thinking aid condition (left panel) and violin plot with box plots of the frequencies of self-administered shocks in each condition (right panel).

Overall, 54 participants (59.3% of the sample; 69% males, 54.8% females) administered shocks to themselves. Figure 5 shows the proportion of participants who administered shocks to themselves in each thinking aid condition. Although the proportion was numerically highest in the unpleasant thinking aid condition and numerically lowest in the pleasant thinking aid condition, this difference was not significant in a Chi-Square test, ${\chi }^{2}$(2, *N* = 91) = 1.14, *p* = 0.566.

The median value of the number of self-administered shocks was *Mdn* = 1 (*IQR* = 8), with large inter-individual differences (range: 229). Participants waited *M* = 29 s (*SD* = 43) before the first keypress. A Kruskal–Wallis H test showed that frequencies of self-administered shocks in the thinking aid conditions were not statistically different, ${\chi }^{2}$(2, *N* = 91) = 0.99, *p* = 0.609. One-sample Wilcoxon signed-rank tests against zero revealed significant self-administration of shocks in the unpleasant (*Mdn* = 2, *IQR* = 9.25), *z* = 3.92, *p* < 0.001, *r* = 0.70, control (*Mdn* = 1, *IQR* = 7), *z* = 3.73, *p* < 0.001, *r* = 0.67, and pleasant thinking aid conditions (*Mdn* = 1, *IQR* = 7.25), *z* = 3.52, *p* < 0.001, *r* = 0.63. Removal of nine outlier values (2 in the pleasant, 4 in the neutral, 4 in the unpleasant thinking aide conditions) did not change these results.

**Figure 6:**
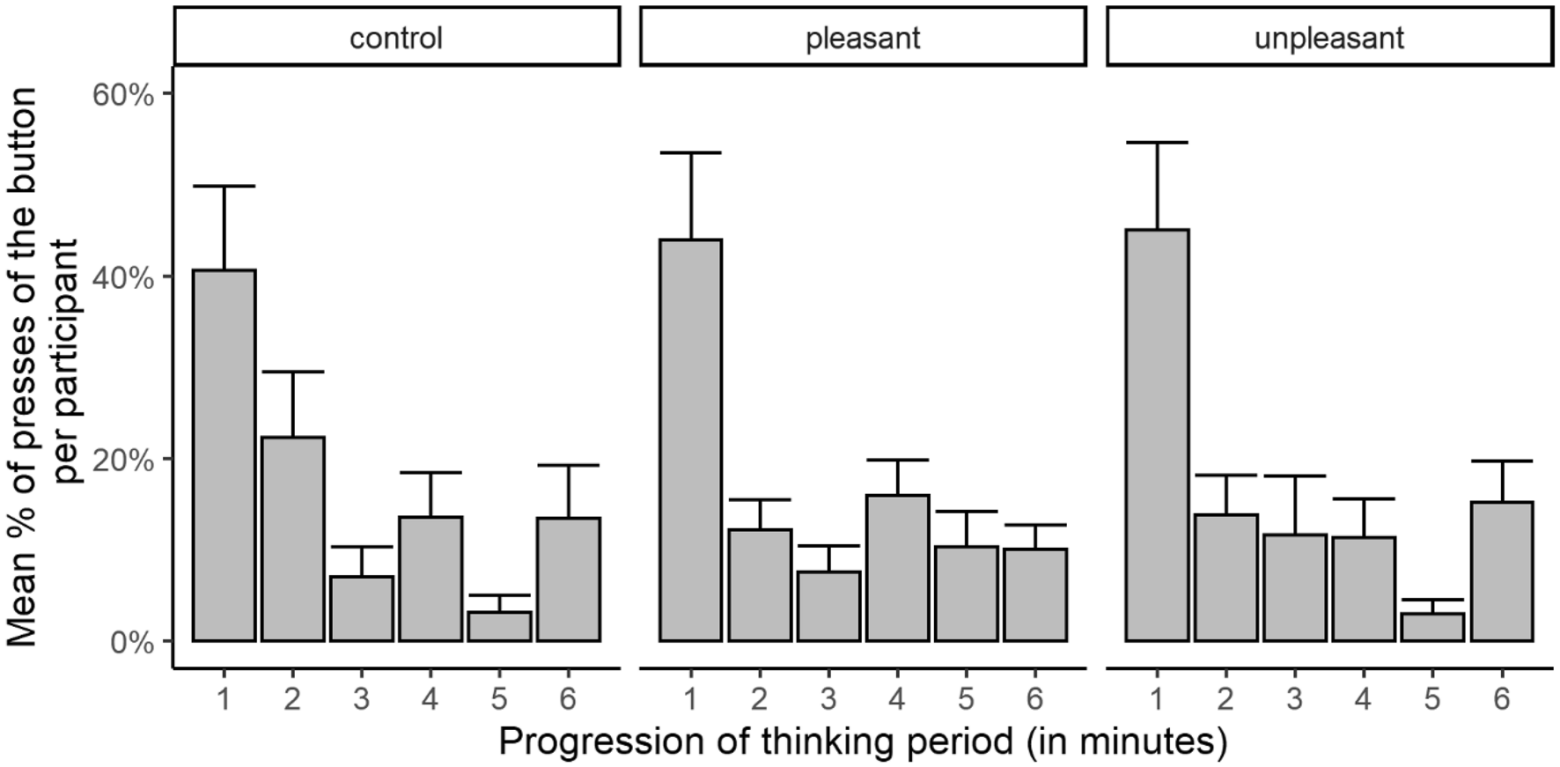
Rate of button presses as a function of time in each thinking aid condition. Error bars show the standard error. Subjects without button presses and nine subjects with outlier values were not included in the counts (valid *n*s = 15, 16, and 14 in the control, pleasant, and unpleasant conditions, respectively).

Figure 6 shows the rate of keypressing as a function of time in each thinking aid condition. Participants waited *M* = 29 s (*SD* = 43) on average before the first keypress. Self-administration of shock was most frequent during the first minutes of the waiting period. Statistical trend analyses of response rates per minute (proportion) in each thinking aide condition in a Bonferroni-adjusted ANOVA with *Waiting Time* (6: each minute of the period, within) and *Thinking Aid Condition* (3: control, pleasant, unpleasant; between) revealed significant linear trends for self-administered shocks in the pleasant thinking aid condition, *F*(1, 13) = 8.83, *p* = 0.033, ${\upeta }_{\mathrm{p}}^{2}$=0.405; unpleasant thinking aid condition, *F*(1, 15) = 8.87, *p* = 0.027, ${\upeta }_{\mathrm{p}}^{2}$=0.372; and in the control condition without thinking aide, *F*(1, 14) = 8.77, *p* = 0.030, ${\upeta }_{\mathrm{p}}^{2}$=0.385. The quadratic trend in the unpleasant thinking aid condition was also significant, *F*(1, 15) = 7.54, *p* = 0.045, ${\upeta }_{\mathrm{p}}^{2}$=0.335. Other trends were not significant with *p*s > 0.05.

#### Ratings of the thinking period

Table 4 shows the ratings. In a 2 × 3 mixed ANOVA of the mood ratings with *Time* (pre, post; within-subjects) and *Thinking Aid Condition* (positive, control, negative; between-subjects), the main effects of *Condition* and *Time* were not significant, *F*(2, 88) = 1.36, *p* = 0.263, ${\upeta }_{\mathrm{p}}^{2}$=0.030, and *F*(1, 88) = 2.73, *p* = 0.102, ${\upeta }_{\mathrm{p }}^{2}$= 0.030, respectively. However, the interaction effect was, *F*(2, 88) = 9.81, *p* < 0.001, ${\upeta }_{\mathrm{p}}^{2}$ = 0.182. Mood improved in the pleasant thinking aid condition and declined in the unpleasant thinking condition, with no change in the neutral condition (see Table 1). Effects of the thinking aid condition on other ratings were tested with one-way ANOVAs. Participants in the unpleasant thinking condition rated the thinking period as more unpleasant, *F*(2, 88) = 6.09, *p* = 0.003, ${\upeta }_{\mathrm{p}}^{2}$= 0.122, and less entertaining *F*(2, 88) = 8.88, *p* < 0.001, ${\upeta }_{\mathrm{p}}^{2}$= 0.168. Ratings of boredom were lowest in the pleasant and highest in the unpleasant thinking conditions; however, this difference was not significant, *F*(2, 88) = 1.82, *p* = 0.168, ${\upeta }_{\mathrm{p}}^{2}$ = 0.040. Ratings of mind-wandering were not affected by pleasant and unpleasant thinking aids, *F*(2, 88) = 0.04, *p* = 0.958, ${\upeta }_{\mathrm{p}}^{2}$= 0.001. Difficulty of engagement in thinking was rated lower in the pleasant thinking aid condition (with one missing questionnaire score due to a technical failure) compared to the control condition and the unpleasant condition, but this difference was not statistically significant, *F*(2, 87) = 1.83, *p* = 0.166, ${\upeta }_{\mathrm{p}}^{2}$ = 0.040.

**Table 4:** Ratings (mean, 95% CI) in the thinking aid conditions.

	Pleasant	Control	Unpleasant
Mood before (−4 to + 4)	1.60 [0.94, 2.26]	1.74 [1.22, 2.26]	1.70 [1.09, 2.31]
Mood after (−4 to + 4)	2.00 [1.47, 2.53]	1.68 [1.16, 2.20]	0.73 [0.34, 1.43]
Pleasantness (−4 to + 4)	1.13 [0.54, 1.73]	1.00 [0.51, 1.49]	-0.13 [-0.77, 0.50]
Entertainment (1 to 9)	5.10 [4.47, 5.73]	5.03 [4.39, 5.68]	3.4 [2.7, 4.10]
Boredom (1 to 9)	4.00 [3.23, 4.77]	4.42 [3.78, 5.06]	5.0 [4.15, 5.85]
Mind-wandering (1 to 9)	5.63 [4.86, 6.40]	5.61 [4.95, 6.27]	5.5 [4.8, 6.2]
Difficulty (1 to 9)	2.69 [2.10, 3.28]	3.58 [2.75, 4.42]	3.6 [2.74, 4.46]

Numbers in round brackets indicate the rating scale from low to high.

Correlational analyses revealed a statistically significant, negative correlation between numbers of self-administrated shocks and mood change (i.e., the fewer self-administrations of shocks, the better the mood after the thinking period). Thinking aid condition was not related to the frequency of self-administered shocks (see Table 5).

**Table 5:** Spearman’s rank-based correlations between thinking aide conditions, mood change, ratings of the thinking period, and counts of self-administered shocks.

	1	2	3	4	5	6	7
1. Thinking aid	–						
2. MOOD	0.397***	–					
3. PLEASANTNESS	0.300**	0.407***	–				
4. BOREDOM	−0.201*	−0.408***	−0.423***	–			
5. ENTERTAINMENT	.0361***	0.470***	0.567***	−0.710***	–		
6. MINDWANDERING	0.048	−0.036	0.079	0.110	0.024	–	
7. DIFFICULTY	−0.136	−0.295**	−0.330**	0.413***	−0.379***	−0.043	–
8. Total count	−0.094	−0.367***	−0.204	0.064	−0.025	−0.022	0.116

Thinking aide condition (−1 = negative, 0 = control, + 1 = positive), *p < .05, **p < .01, ***p < .001.

### Discussion

Thinking aides were effective in prompting pleasant and unpleasant thoughts during the thinking period: participants in the unpleasant thinking condition rated the thinking period as more unpleasant and less entertaining compared to participants in the pleasant thinking condition. Assistance in the generation of (un)pleasant thoughts had however no statistically significant effect on the self-administration of electric shocks. This null finding adds another piece of evidence to the conclusion that escape from negative thought is not the exclusive, or primary, motivational reasons for the self-administration of electric shocks.

## General discussion

Why would people favor electric shocks over idly sitting in a room, having nothing but their thoughts? For Wilson and colleagues, the reason was the discomfort produced by the difficulty to engage in directed pleasurable thoughts^1–3^. According to their explanation, deliberate thinking for pleasure is effortful because it requires both motivation and the ability to concentrate. Therefore, most people prefer to distract themselves when being left alone with their thoughts, because that distraction is less discomforting than engaging in thoughts.

The present studies confirm the previous finding that many people would rather administer shocks to themselves than be left alone with their thoughts^2^. Most participants in our studies opted to administer shocks to themselves (range: 63–89%), and proportions were substantially larger than in the original study (43%). We were surprised by these large effects because the intensity of the shock was carefully adjusted in our studies to be slightly painful for each individual—and hence likely stronger than that used in the study of Wilson and colleagues.

The present research however does not provide strong support for an explanation with negative reinforcement. According to this account, people administer shocks to themselves because the painful stimulation is less aversive than the engagement in the thinking task^1,2^. This avoidance orientation implies that participants should have opted for the least aversive stimulation in their search for distraction, which was not observed. In fact, a majority opted for strong shocks when they could select a mild shock (Studies 1–3), and they shocked themselves when they had an innocuous response option without shock administration available for distraction (Study 3). A large number also shocked themselves when they were aided in the generation of entertaining thoughts, and it made no difference whether the contents of their thoughts were pleasant or unpleasant (Study 4). These findings are clearly at odds with the negative reinforcement account, according to which people should opt for the least aversive stimulation in their search for distraction.

Our data show that participants knowingly administered intense shocks to themselves although they had less painful alternatives available. One explanation for this behavior could be that participants administered shocks to themselves as for a distraction from the thinking task, and that they believed that intense shocks would better serve this purpose. This explanation would still maintain that intentional engagement in thinking was unpleasant or at least not entertaining for participation; it would however question the assumption that minimization of unpleasant affect was the exclusive motivational reason for the self-infliction of pain. If the main purpose of self-administration of shocks was distraction, participants should prefer activities during a waiting period that have the highest expectation to provide a distraction, irrespective of their intrinsic pleasantness. This means painful shocks were not administered because they were less unpleasant than engagement in thinking but, rather, because they were more entertaining. Future research could examine this hypothesis by giving participants a choice between unpleasant activities that differ in their potential to provide a distraction while triggering unpleasant affects to a similar degree.

Participants could have also searched for the thrill of (intense) electric shocks because the self-stimulation disrupted the monotony of the waiting period^5,6^. This explanation is partially supported by significant positive correlations between numbers of self-administered shocks and ratings of boredom and/or excitement in Studies 1a and 1b; however, no positive correlations were observed in the other studies (see the supplement), which questions the robustness of this relationship. Furthermore, participants did not systematically prefer administrations of strong shocks or those with random intensities, which were arguably the most exciting response options. Trend analyses of shock administrations over time in Study 3 and 4 also revealed that shock administrations were most frequent at the beginning of the waiting period and declined during the waiting period, which is difficult to reconcile with the disruption of a boredom state induced by waiting.

Another possibility is that the self-administration of electric shocks itself was attractive . What could have made the self-infliction of pain attractive? First, it should be noted that when given a choice, we did not observe a clear preference for a particular shock type that would have been indicative of negative reinforcement (no/mild shocks), thrill seeking (strong shock), or curiosity (uncertain/random shock intensity). In contrast, a large majority administered all four shock types to themselves, which could be seen as an explorative behavior. Exploration could explain why a larger proportion of participants opted to shock themselves in Study 1 compared to Study 2, in which they could explore each shock intensity before the thinking period. Furthermore, many participants expressed in post-experimental interviews that they administered shocks to themselves because they were curious, for instance, about the feelings of two different shocks feel or because they mistrusted the task setup (for a report see the supplementary information file). Thus, it appears that a large proportion used the shock device because they were curious about the shocks. Many participants also administered shocks to themselves multiple times, which could be seen as a testing behavior how many shocks one could endure or how the pain feelings would change after multiple administrations. Limit testing would also explain why a small, but consistent fraction in each experiment administered hundreds of electric shocks to themselves, which is an extreme behavior that was also observed by Wilson and colleagues^2^.

To summarize, the present findings question that the exclusive motivation for the self-administration of shocks in our research, and presumably also in the research of Wilson and colleagues^2^, was avoidance of thinking and the associated minimization of negative affect. Instead, our data suggest that the self-infliction of pain may have been attractive for many participants, because they were curious about the shocks, their intensities, and the effects they would have on them (limit testing). This interpretation does of course not suggest that engagement in thinking is pleasant. To the contrary, Wilson and colleagues^1^ summarized many circumstances in which people preferred other activities over thinking, which highlights that deliberate thinking for pleasure was not ranked particularly high by most people. However, the mere observation that one activity was preferred over another activity does not logically imply that the non-preferred activity was unpleasant^4^; this means researchers should refrain from assigning absolute values or hedonic categories to activities and events (for elaborate discussions see^22,23^). A categorization fallacy also applies to allegedly ‘unpleasant’ activities, such as the self-administration of painful shocks, which could be attractive, despite generating unpleasant sensations, because it could still a particular human need (e.g., curiosity). It is well-documented that people often strive to resolve ambiguities even if it is risky and without apparent instrumental benefit^8,24,25^. People are curious about high intensity negative information, being fascinated by media coverages of intense violence or by horror movies^7,26^, and neuroimaging studies showed that satisfaction of one’s curiosity activates reward-related circuitries in the brain, even when the information search exposes the individual to unpleasant stimuli^27,28^. The present studies are in line with this research, highlighting that people are willing to leave their comfort zone for a new experience.

## Supplementary Information

Supplementary Information.

## Data Availability

Raw data underlying the main findings reported in this paper can be accessed at https://doi.org/10.7910/DVN/IQHACM.

## References

[1] Wilson, T. D., Westgate, E. C., Buttrick, N. R. & Gilbert, D. T. *Advances in Experimental Social Psychology* (ed. Olson, J. M.). Vol. **60**. 175–221. (Academic Press, 2019).

[2] Wilson TD (2014). Just think: The challenges of the disengaged mind. Science.

[3] Alahmadi S (2017). You can do it if you really try: The effects of motivation on thinking for pleasure. Motiv Emot.

[4] Fox, K. C. R., Thompson, E., Andrews-Hanna, J. R. & Christoff, K. Is thinking really aversive? A commentary on Wilson et al.’s “Just think: the challenges of the disengaged mind”. *Front Psychol* **5,** 1497 (2014). 10.3389/fpsyg.2014.01427 PMC4260464 25538668

[5] Nederkoorn C, Vancleef L, Wilkenhöner A, Claes L, Havermans RC (2016). Self-inflicted pain out of boredom. Psychiatry Res..

[6] Havermans RC, Vancleef L, Kalamatianos A, Nederkoorn C (2015). Eating and inflicting pain out of boredom. Appetite.

[7] Zuckerman M, Litle P (1986). Personality and curiosity about morbid and sexual events. Pers. Individ. Differ..

[8] Hsee CK, Ruan B (2016). The Pandora effect: The power and peril of curiosity. Psychol. Sci..

[9] Brysbaert M (2019). How many participants do we have to include in properly powered experiments? A tutorial of power analysis with reference tables. J. Cogn..

[10] Faul F, Erdfelder E, Lang A-G, Buchner A (2007). G*Power 3: A flexible statistical power analysis program for the social, behavioral, and biomedical sciences. Behav. Res. Methods.

[11] Eder AB, Dignath D, Erle TM, Wiemer J (2017). Shocking action: Facilitative effects of punishing electric shocks on action control. J. Exp. Psychol. Gen..

[12] Crosbie, J. *Handbook of Research Methods in Human Operant Behavior.* (eds. Lattal, K. A. & Perone, M.). 163–189. (Plenum Press, 1998).

[13] Church RM, Raymond GA, Beauchamp RD (1967). Response suppression as a function of intensity and duration of a punishment. J. Comp. Physiol. Psychol..

[14] Satow, L. *B5T—Psychomeda Big-Five-Persönlichkeitstest [B5T—Psychomeda Big Five Personality Inventory]*. <Elektronisches Testarchiv PSYNDEX> (Elektronisches Testarchiv, 2011).

[15] Bless H, Wänke M, Bohner G, Fellhauer RF, Schwarz N (1994). Need for cognition: A scale measuring engagement and happiness in cognitive tasks [German Version]. Z. Sozialpsychol..

[16] Roth M, Hammelstein P (2011). The need inventory of sensation seeking (NISS) [German version]. Eur. J. Psychol. Assess..

[17] Mummendey, H. D. & Eifler, S. *Ein Fragebogen zur Erfassung ‘Positiver’ Selbstdarstellung (Impression-Management Skala) [Questionnaire for the Measurement of Impression Management].* https://pub.uni-bielefeld.de/download/2462388/2604057/170_Mummendey_Fragebogen.pdf (University Bielefeld, 1994).

[18] von Collani, G. *Modifizierte Deutsche Versionen des Narcissistic Personality Inventory (NIP-d)*. 10.6102/zis51 (GESIS-Leibniz-Institut für Sozialwissenschaften, 2014).

[19] Konrath S, Meier BP, Bushman BJ (2014). Development and validation of the Single Item Narcissism Scale (SINS). PLoS ONE.

[20] Tukey JW (1977). Exploratory Data Analysis.

[21] Westgate EC, Wilson TD, Gilbert DT (2017). With a little help for our thoughts: Making it easier to think for pleasure. Emotion.

[22] Flaherty CF (1996). Incentive Relativity.

[23] Parducci, A. *Attitudinal Judgment* (ed. Eiser, J. R.). 3–21. 10.1007/978-1-4613-8251-5_1 (Springer, 1984).

[24] Litman J (2005). Curiosity and the pleasures of learning: Wanting and liking new information. Cogn. Emot..

[25] Kruger J, Evans M (2009). The paradox of Alypius and the pursuit of unwanted information. J. Exp. Soc. Psychol..

[26] Oosterwijk S (2017). Choosing the negative: A behavioral demonstration of morbid curiosity. PLoS ONE.

[27] Lau JKL, Ozono H, Kuratomi K, Komiya A, Murayama K (2020). Shared striatal activity in decisions to satisfy curiosity and hunger at the risk of electric shocks. Nat. Hum. Behav..

[28] Oosterwijk S, Snoek L, Tekoppele J, Engelbert LH, Scholte HS (2020). Choosing to view morbid information involves reward circuitry. Sci. Rep..

