# Supplementary material for: Motivations underlying self-infliction of pain during thinking for pleasure
Source: Sci Rep. 2022 Jul 4;12:11247. doi: 10.1038/s41598-022-14775-w (PMC9253005; doi:10.1038/s41598-022-14775-w)
Supplement: Supplementary file 1 — Supplementary Information. [file 41598_2022_14775_MOESM1_ESM.docx]

**Supplementary Information File**

****Gender differences (*n* = 254)****

Data across the four studies were aggregated for an analysis of gender differences in the self-administration of shocks (81 men, 173 women). Proportions of men (77.8%) and women (75.1%) that opted to administer a shock to themselves during the thinking period were not statistically different, $\chi^{2}$(1, *N* = 254) = 0.21, *p* = .647. Furthermore, men and women administered shocks to themselves with similar frequencies (*Mdn* = 9 *vs* 7), *U* = 6307, *z* = -1.29, *p* = .196. Comparisons for each shock intensity with Mann-Whitney U tests revealed no gender differences in the median frequencies of administered mild shocks, *U* = 2635.5, *z* = -0.896, *p* = .370, medium shocks, *U* = 2765.5, z = -0.432, *p* = .666, strong shocks, *U* = 2517, *z* = -1.329, *p* = .184, and shocks with random intensities, *U* = 1420, *z* = -1.50, *p* = .134.

In addition to an overall analysis, we also analyzed gender differences for each study. Note, however, that the interpretation of these results is compromised by the uneven distributions of male and female participants across studies (for numbers see our main report). Proportions of men and women that opted to administer a shock to themselves during the thinking period were not statistically different in Study 1, $\chi^{2}$(1, *N* = 70) = 0.88, *p* = .766; Study 2, $\chi^{2}$(1, *N* = 59) = 0.42, *p* = .519; Study 4, $\chi^{2}$(1, *N* = 91) = 1.63, *p* = .201. In Study 3, more males relative to females opted to administer shocks to themselves $\chi^{2}$(1, *N* = 34) = 5.74, *p* = .017; however, this difference should be interpreted with caution because the sample size of this study (15 males and 19 females) was the smallest in our set of studies. In respect to the total numbers of shock administrations, there were no significant gender differences in a study (with *ps* ≥ .080).

**Study 1 (qualitative analysis)**

We also analyzed participants’ answers to the open-ended questions that inquired the reasons for a self-administration of shocks. Written answers from 58 participants were available for this analysis. The answers were sorted by a research assistant blind to the research hypotheses into pre-defined categories (e.g., averseness of the situation, curiosity, distrust, anxiety), and the sorting was independently rechecked by a researcher. A majority of participants (69%) indicated that they administered shocks for reasons of curiosity, for instance, for a comparison of the intensities of two different shocks. Other participants indicated that they made a game of predicting the intensity of the random shock. Another large answer cluster (38%) openly expressed mistrust of the task setup and/or checking behavior. Notably, only a single participant stated expressis verbis that aversive thinking was the reason for a self-administration of shocks.

Studies 1 and 2 (*n* = 128, one missing questionnaire)

Table S1

*Spearman’s rank-based correlations between personality measures and frequencies of self-administered shocks*

|  | ***1*** | | ***2*** | | ***3*** | | ***4*** | | ***5*** | | ***6*** | | ***7*** | | ***8*** | | **9** |
| --- | --- | --- | --- | --- | --- | --- | --- | --- | --- | --- | --- | --- | --- | --- | --- | --- | --- |
| 1. Openness | — |  |  |  |  |  |  |  |  |  |  |  |  |  |  |  |  |
| 2. Neuroticism | -.101 |  | — |  |  |  |  |  |  |  |  |  |  |  |  |  |  |
| 3. nAch | -.710 | *** | .026 |  | — |  |  |  |  |  |  |  |  |  |  |  |  |
| 4. NFC | .035 |  | -.195 | * | .210 | * | — |  |  |  |  |  |  |  |  |  |  |
| 5. NISS-NS | .822 | *** | -.030 |  | -.721 | *** | -.124 |  | — |  |  |  |  |  |  |  |  |
| 6. NISS-AR | .797 | *** | -.005 |  | -.734 | *** | -.083 |  | .815 | *** | — |  |  |  |  |  |  |
| 7. IMS | .577 | *** | -.039 |  | -.329 | *** | .038 |  | .526 | *** | .495 | *** | — |  |  |  |  |
| 8. NPI-d | -.668 | *** | -.011 |  | .794 | *** | .074 |  | -.695 | *** | -.688 | *** | -.349 | *** | — |  |  |
| 9. SINS | -.675 | *** | .056 |  | .828 | *** | .067 |  | -.662 | *** | -.689 | *** | -.344 | *** | .819 | *** | — |
| Mild | .091 |  | .123 |  | .051 |  | .088 |  | .075 |  | .083 |  | .130 |  | .035 |  | .096 |
| Medium | .012 |  | .151 |  | .122 |  | .070 |  | -.034 |  | -.008 |  | .061 |  | .114 |  | .180 |
| Strong | .027 |  | .125 |  | .119 |  | .091 |  | -.016 |  | .022 |  | .055 |  | .076 |  | .171 |
| Random | -.028 |  | .112 |  | .145 |  | .079 |  | -.010 |  | -.027 |  | .042 |  | .121 |  | .174 |
| Total count | .042 |  | .149 |  | .106 |  | .079 |  | .009 |  | .029 |  | .101 |  | .081 |  | .148 |

Note. * *p* < .05, ** *p* < .01, *** *p* < .001
